# The Hygienic Importance of Amusements

**Published:** 1896-06

**Authors:** 


### The Hygienic Importance of Amusements.

At the recent annual banquet of the French Societe de Hygiene, one speaker dwelt upon the absolute necessity of providing amusements for the masses as a hygienic measure. This was better understood in the past than now, and entertainments were provided for the people by the State. He mentioned an instance in his own experience, of a regiment whose commanding officer allowed his men to sing on the march. Health, spirits and strength flourished, and the severest exercises were play to the light-hearted men. The officer was changed and the new-comer stopped the singing, when the men drooped and the sick list grew long.

